# Editorial: Mitochondria as a hub for neurodegenerative disorders

**DOI:** 10.3389/fnmol.2023.1147468

**Published:** 2023-02-06

**Authors:** Verena Kohler, Ralf J. Braun, Andreas Aufschnaiter

**Affiliations:** ^1^Institute of Molecular Biosciences, University of Graz, Graz, Austria; ^2^Department of Molecular Biosciences, The Wenner-Gren Institute, Stockholm University, Stockholm, Sweden; ^3^Research Division for Neurodegenerative Diseases, Center for Biosciences, Department of Medicine, Faculty of Medicine and Dentistry, Danube Private University, Krems an der Donau, Austria; ^4^Department of Biochemistry and Biophysics, Stockholm University, Stockholm, Sweden

**Keywords:** mitochondria, neurodegeneration, protein homeostasis, cell death, oxidative stress, aging, neuronal disease

Mitochondria are vital organelles best known for their function in cellular energy conversion *via* oxidative phosphorylation (OXPHOS). However, they conduct a plethora of physiological functions, ranging from central metabolic activities, ion homeostasis, and proteostasis, to regulation of cellular proliferation and cell death. To adapt to these important physiological tasks according to cellular needs, mitochondria exist as an extended dynamic network, interacting with the vast majority of other cellular organelles (Harper et al., 2020). Considering the complexity of mitochondrial morphology and functions, it comes as no surprise that disturbances of these organelles have deleterious consequences for cellular health. Neurons are particularly vulnerable to mitochondrial malfunction due to their high energy demand, and hence, mitochondrial dysfunction is a prerequisite for many neurodegenerative diseases (Aufschnaiter et al., 2017). However, the molecular details on this complex relationship between mitochondrial malfunction and specific alterations of neurons resulting in neurodegeneration remain poorly understood. The articles in this Research Topic explored the intertwined molecular connections between mitochondrial dysfunction and neurodegeneration.

A unique feature of mitochondria is the presence of their own genome in the form of mitochondrial DNA (mtDNA). Hence, separate and specialized molecular machineries for genome maintenance, gene expression, and protein biosynthesis are required inside mitochondria to guarantee functionality of these organelles (Ott et al., 2016). As this genome encodes essential proteins for the OXPHOS machinery, a sophisticated interplay and tight coordination with the gene expression of nuclear-encoded OXPHOS components is required to maintain cellular energy conversion. Yao et al. addressed the intriguing question of whether multi-mtDNA variants can be a factor contributing to mitochondrial function variety in Leber's Hereditary Optic Neuropathy (LHON). Indeed, the authors found that cells with more mtDNA variants had increased levels of reactive oxygen species and reduced OXPHOS functionality. A disruption of respiration was also found by Trease et al., who analyzed the consequences of hyperphosphorylated human tau in a mouse model of tauopathy. Interestingly, human tau preferentially bound to synaptic mitochondria, thereby not altering synaptosomal mitochondrial content or basal mitochondrial respiration, but rather leading to impairment of maximal mitochondrial respiration, potentially adding to the observed synaptic dysfunctions.

Not least due to defects in mitochondrial respiration and the resulting high levels of oxidative stress, mitochondrial protein quality control systems constantly need to monitor and maintain mitochondrial protein homeostasis. Jishi and Qi discussed mechanisms of mitochondrial proteostasis and their alterations in neurodegenerative disorders. In case molecular damages inside mitochondria cannot be prevented or repaired by respective quality control systems, whole organelles can also be removed by a selective form of autophagy, termed mitophagy. Wang Q. et al. discussed the possibility to counteract neurodegenerative disorders by pharmacologically enhancing mitophagy. Mitochondrial dynamics is essential for this selective form of mitochondrial degradation, as damaged parts of mitochondria need to be separated. However, these processes can also be impaired in neurodegenerative processes. Thorne et al. reviewed such dysfunctions of mitochondrial dynamics in the pathogenesis of Parkinson's disease, focusing on malfunctions caused by α-synuclein. The authors thereby also highlighted the necessity to study the functional implications of α-synuclein in these processes to gain mechanistic insights into both the physiological and pathophysiological roles of this protein in mitochondrial dynamics and quality control.

Another risk locus for Parkinson's disease is gene coding for the outer mitochondrial membrane Rho GTPase Miro1. This protein is not only involved in mitophagy, but also in proper distribution of mitochondria to synapses and maintenance of calcium homeostasis. The mutation R272Q within the calcium binding domain of Miro1 is found in patients with sporadic Parkinson's disease. Schwarz et al. demonstrated that this mutation disrupts mitochondrial calcium handling *via* events at the outer mitochondrial membrane. Interestingly, even though affected mitochondria displayed fragmentation and altered cristae organization, neither CCCP-induced mitophagy nor mitochondrial movement were affected by this variant.

Neurodegenerative processes trigger an avalanche of phenotypes, which often makes it difficult to characterize causal molecular mechanisms of cellular dysfunction. Time-resolved studies can provide important insights into the sequence of molecular events and thus, present an important tool for characterizing the pathophysiology of age-dependent diseases. Wang S. et al. performed such an analysis with various mutants of the amyloid precursor protein (APP) in mice. The authors showed that mitochondrial dysfunction preceded behavioral changes, suggesting mitochondrial dysfunction as an early event in the pathogenesis of Alzheimer's disease.

Overall, this Research Topic collected original articles showcasing novel aspects and molecular mechanisms of altered mitochondrial function in neurodegenerative disorders, and timely review articles discussing the latest findings on mitochondrial protein homeostasis, protein quality control, and dynamics in neurodegenerative diseases. The broad range of articles provides a comprehensive perspective on mitochondrial dysfunction in neurological disorders, highly interesting for biologists and clinicians alike.

## Author contributions

AA wrote the primary draft. VK, RB, and AA edited and finalized the article. All authors contributed to the article and approved the submitted version.
